# Transcranial Direct Current Stimulation Combined With Listening to Preferred Music Alters Cortical Speech Processing in Older Adults

**DOI:** 10.3389/fnins.2022.884130

**Published:** 2022-07-06

**Authors:** Gavin M. Bidelman, Ricky Chow, Alix Noly-Gandon, Jennifer D. Ryan, Karen L. Bell, Rose Rizzi, Claude Alain

**Affiliations:** ^1^Department of Speech, Language and Hearing Sciences, Indiana University Bloomington, Bloomington, IN, United States; ^2^School of Communication Sciences and Disorders, The University of Memphis, Memphis, TN, United States; ^3^Rotman Research Institute, Baycrest Centre, Toronto, ON, Canada; ^4^Department of Psychology, University of Toronto, Toronto, ON, Canada; ^5^Department of Psychiatry, University of Toronto, Toronto, ON, Canada; ^6^Institute of Medical Science, University of Toronto, Toronto, ON, Canada; ^7^Department of Audiology, San José State University, San Jose, CA, United States; ^8^Music and Health Science Research Collaboratory, University of Toronto, Toronto, ON, Canada

**Keywords:** aging, brain stimulation, event-related potential (ERP), frequency-following response (FFR), speech-in-noise (SIN) perception

## Abstract

Emerging evidence suggests transcranial direct current stimulation (tDCS) can improve cognitive performance in older adults. Similarly, music listening may improve arousal and stimulate subsequent performance on memory-related tasks. We examined the synergistic effects of tDCS paired with music listening on auditory neurobehavioral measures to investigate causal evidence of short-term plasticity in speech processing among older adults. In a randomized sham-controlled crossover study, we measured how combined anodal tDCS over dorsolateral prefrontal cortex (DLPFC) paired with listening to autobiographically salient music alters neural speech processing in older adults compared to either music listening (sham stimulation) or tDCS alone. EEG assays included both frequency-following responses (FFRs) and auditory event-related potentials (ERPs) to trace neuromodulation-related changes at brainstem and cortical levels. Relative to music without tDCS (sham), we found tDCS alone (without music) modulates the early cortical neural encoding of speech in the time frame of ∼100–150 ms. Whereas tDCS by itself appeared to largely produce suppressive effects (i.e., reducing ERP amplitude), concurrent music with tDCS restored responses to those of the music+sham levels. However, the interpretation of this effect is somewhat ambiguous as this neural modulation could be attributable to a true effect of tDCS or presence/absence music. Still, the combined benefit of tDCS+music (above tDCS alone) was correlated with listeners’ education level suggesting the benefit of neurostimulation paired with music might depend on listener demographics. tDCS changes in speech-FFRs were not observed with DLPFC stimulation. Improvements in working memory pre to post session were also associated with better speech-in-noise listening skills. Our findings provide new causal evidence that combined tDCS+music relative to tDCS-alone (i) modulates the early (100–150 ms) cortical encoding of speech and (ii) improves working memory, a cognitive skill which may indirectly bolster noise-degraded speech perception in older listeners.

## Introduction

Transcranial direct current stimulation (tDCS) is a type of neuromodulation technique that exerts effects over a specified cortical target region of the brain. Anodal and cathodal electrodes are placed on the scalp and low intensity direct current is applied resulting in polarity-dependent changes in cortical activity (Neuling et al., 2012). Though the mechanisms by which tDCS-modulated brain activity are debated (Stagg et al., 2009), it is thought anodal stimulation elicits depolarization of neurons in cortical target regions, increasing the probability of action potentials, whereas cathodal stimulation acts to hyperpolarize surrounding membrane potentials thereby decreasing the likelihood of neuronal firing (Stagg et al., 2009; Thair et al., 2017; Wang et al., 2020). Given its ability to upregulate/downregulate neuronal signaling, tDCS is being actively explored as a means to enhance behavioral functions via selected targeting of brain areas associated with certain perceptual-cognitive processes.

Transcranial direct current stimulation studies have overwhelmingly focused on improving memory functions (for review, see Thair et al., 2017). Fewer studies have examined neuroplastic effects of tDCS on aspects of audition and sensory processing (cf. Antal et al., 2004; Bolognini et al., 2011; Castaño-Castaño et al., 2019). Generally in these studies, 1–2 mA of direct current is applied over auditory cortex for 10–20-min intervals (Wang et al., 2020). Changes in brain activity can then be tracked non-invasively via scalp-recorded electroencephalography (EEG) and event-related brain potentials (ERPs). Using these techniques, two studies found that tDCS influenced the strength of the auditory P50 (frontocentral positive wave peaking at ∼50 ms), suggesting tDCS can modulate auditory stimulus gating (Zaehle et al., 2011; Terada et al., 2015). However, Kunzelmann et al. (2018) noted that electrode location, current intensity, and stimulation interval have dramatic effects on tDCS effects and corresponding neural responses, which may account for the highly variable and subtle effects of neurostimulation on auditory perception reported in prior work.

Nevertheless, a handful of studies have shown positive effects of tDCS on auditory processing. For instance, cathodal stimulation over auditory temporal cortex improves phonetic categorization and enhanced the amplitude of the P50 ERP in response to consonant vowel sounds (Heimrath et al., 2016). Anodal tDCS has also been used to modulate auditory stream segregation (Deike et al., 2016) and improve performance on sound discrimination tasks (Ladeira et al., 2011; Impey and Knott, 2015). Both positive and negative results have been reported for pitch discrimination tasks with stimulation over Heschl’s gyrus (Mathys et al., 2010; Matsushita et al., 2021). For instance, Matsushita et al. (2021) showed that pitch discrimination learning was disrupted when anodal was applied over the right (but not left) auditory cortex. Mixed results have been reported in other sensory domains including vision (Antal et al., 2004; Bolognini et al., 2011; Castaño-Castaño et al., 2019). In relation to speech perception, cathodal tDCS over right inferior frontal gyrus (i.e., right homolog of canonical Broca’s area) increases prosodic comprehension during dichotic listening (Alexander et al., 2012). Thus, while there is some discrepancy on the direction of tDCS effects (i.e., enhancement or suppression of brain function) and whether anodal vs. cathodal stimulation is more effective, the mere presence of these neuromodulatory effects on auditory brain activity demonstrates tDCS can be used to identify causal relationships between stimulating neural pathways inducing neuroplasticity and behavior (Boroda et al., 2020). Although tDCS may be effective for alleviating some deficits in aberrant cognitive aging (e.g., dementias; Hsu et al., 2015), it is not yet clear whether such benefits extend to the healthy aging population (Nilsson et al., 2015; Berryhill and Martin, 2018). If tDCS does facilitate neuroplasticity and influence perceptual processing, then it could be offered in clinical settings to enhance or at least offset declines in receptive hearing abilities that normally decline with age.

Listening to music is another potential activity that may aid perceptual-cognitive functions. Indeed, enjoyable music can heighten arousal and induce positive affect, thereby influencing subsequent cognitive performance (Thompson et al., 2001; Husain et al., 2002). Autobiographically salient music (i.e., music evoking salient personal memories), in particular, can evoke strong positive affect and heighten arousal (Belfi et al., 2016). In older adults, music listening can boost various cognitive domains related to memory including autobiographical memory recall (El Haj et al., 2015), working memory (WM) (Chow et al., 2021), and semantic memory (Bottiroli et al., 2014). Relative to our interest in auditory processing, both long- and short-term music engagement in older adults has also been associated with neural and behavioral improvements in sensory and speech processing (e.g., Parbery-Clark et al., 2012; Alain et al., 2014, 2019; Bidelman and Alain, 2015). In line with the arousal-and-mood hypothesis (Thompson et al., 2001; Husain et al., 2002), we have proposed that listening to autobiographically salient music may have the potential to improve short-term performance in certain cognitive domains, including speech processing, by improving positive affect and heightening arousal state (Chow et al., 2021).

To this end, we recently investigated the synergistic effects of brief (20 min) tDCS and autobiographically salient music listening on cognitive skills in healthy older adults (Chow et al., 2021). EEGs recorded during word recognition recall tracked changes in brain function following tDCS neurostimulation over dorsolateral prefrontal cortex (DLPFC). DLPFC was chosen as the stimulation site to investigate improvements in performance for WM and episodic memory tasks as suggested by prior tDCS studies (Berryhill and Jones, 2012; Manenti et al., 2013). We found changes in several neural signatures underlying recognition memory following tDCS+Music compared to sham stimulation. Additionally, we found improvements in backward memory span and emotional affect under the paired tDCS+Music stimulation. Our findings suggested that listening to autobiographically salient music amplifies the effects of tDCS on cognitive skills and corresponding brain function. Yet, whether the synergistic effects of combined tDCS and music similarly enhance older adults’ auditory speech processing was not formally evaluated.

Here, we report on new EEG data from the sample of older adults reported in Chow et al. (2021). We aimed to determine whether anodal tDCS over DLPFC alters the brain’s early auditory sensory processing of speech. DLPFC is considered a higher-order (non-auditory) brain region and was targeted in previous study to impact memory-related functions. However, several lines of evidence suggest DLPFC might also exert influences on auditory-sensory processing. For example, direct connections between prefrontal and primary auditory cortex have been identified in neuroanatomical tracing studies (Hackett et al., 1999; Plakke and Romanski, 2014). Lesion and non-invasive imaging studies in humans and other mammals also demonstrate that frontal areas modulate auditory activity in superior temporal gyrus (Knight et al., 1989; Fritz et al., 2010; Price et al., 2019; Bidelman and Myers, 2020) including the inputs to primary auditory cortex (Knight et al., 1989). Based on these findings, we reasoned tDCS stimulation over DLPFC might causally impact sound processing in early auditory cortex (and perhaps even earlier in auditory brainstem) as measured by auditory ERPs. In addition, given the strong links between WM skills and speech processing (particularly in noise; Füllgrabe and Rosen, 2016a; Vermeire et al., 2019; Yeend et al., 2019), we reasoned that if tDCS indeed modulates WM performance (cf., Chow et al., 2021), this might relate to older adults’ performance on clean and noise-degraded speech processing tasks.

To this end, we measured frequency-following responses (FFRs) and auditory ERPs to trace tDCS-related changes at both brainstem (FFRs) and cortical (ERPs) levels of the auditory system. We also focused on auditory behavioral measures including hearing thresholds, speech-in-noise processing (QuickSIN) (Killion et al., 2004), and WM (digit span) performance, as these factors are important determinants of day-to-day speech recognition abilities in older adults (e.g., Killion et al., 2004; Moore et al., 2014; Füllgrabe and Rosen, 2016a,b; Bidelman et al., 2019; Vermeire et al., 2019; Yeend et al., 2019). We recorded speech-evoked ERPs after anodal tDCS was concurrently administered to participants listening to autobiographically salient music. These measures were compared to those recorded either after sham stimulation with music listening, or after tDCS administered in silence (Chow et al., 2021). We hypothesized that anodal tDCS paired with music listening would amplify speech-evoked neural responses, providing causal evidence for short-term neuroplasticity in speech processing among older adults.

## Materials and Methods

### Participants

The sample is identical to that reported in our companion paper on the effects of neurostimulation on cognitive processing in older adults (Chow et al., 2021). Results of tDCS and music listening on recognition memory, WM, self-reported ratings of emotional affect [measured by the Positive Affect and Negative Affect Schedule (PANAS)] (Watson et al., 1988), and cognitive ERPs related to word recognition were reported previously (Chow et al., 2021). Here, we investigated the impact of tDCS and music stimulation on speech-evoked ERPs and auditory-related behavioral measures (i.e., audiometric thresholds, speech-in-noise perception; auditory verbal WM).

Briefly, participants were older adults (*N*=14; 72.6 ± 5.03 years; 11 females) who reported normal or corrected-to-normal vision and hearing, with no history of neuropsychiatric illness or disorder. This sample size was similar to those of past tDCS and music listening studies (Picazio et al., 2015; Mansouri et al., 2017). Additionally, we used a within-subject design so that all subjects participated in each type of session, and optimizing the possibility of detecting tDCS effects. All participants were right-handed, had a collegiate level of education (16.8 ± 4.4 years), and had no prior experience with tDCS or any of the experimental tasks. Audiometric testing indicating normal hearing bilaterally [average pure-tone audiometric (PTA) thresholds (500, 1,000, and 2,000 Hz): 17.0 ± 5.8 dB HL]. Details of other exclusion criteria are provided in Chow et al. (2021). Individuals were recruited from the participant database at the Rotman Research Institute as well as local advertisements. Written informed consent was obtained in compliance with a protocol approved by the Research Ethics Board of the Rotman Research Institute at Baycrest Centre. All participants were informed about the procedures and possible risks of tDCS prior to starting and received monetary compensation for their time.

### Pre-experiment Musical Listening Interview

Prior to study participation, participants were asked about musical listening preferences and past musical experiences to ensure autobiographical saliency of the music to be played during the study (Chow et al., 2021). During the interview, participants were probed for their preferred genres of music and asked to share their favorite songs and artists from their past and present. Participants were also probed for music listening preferences throughout each decade of their lifespan starting from childhood and teenage years, and for specific songs (both song names and their respective artists) that held considerable idiosyncratic meaning or evoked personal memories of the past. This information was then used to compile a personalized playlist of songs for each participant lasting ∼21 min (i.e., the duration of the tDCS stimulation phase). Specific songs that were probed through the interview were prioritized for inclusion in each playlist, and the remaining time allotted to the playlist included songs that represented their favorite musical artists and genres from their past. Each playlist thus contained both participant-selected music and music selected by experimenters based on participants’ individual preferences. Playlists were curated from Spotify. Where feasible, study recordings were selected over live versions of each song. Songs varied in time period and musical genre, spanning classical, rock, jazz, folk, pop, country, and film scores. Playlists included both vocal and instrumental-only songs, although most were vocal in nature.

### Experimental Design and Procedure

**FIGURE 1:**
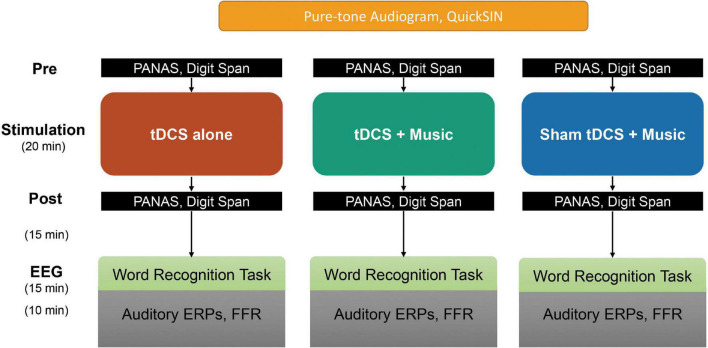
Summary of experimental protocol and measures. Participants first completed audiometric and speech-in-noise testing. They then participated in pre- and post-test behavioral and tDCS sessions, followed by EEG recording. Note that all participants cycled through each of the three tDCS session types separated by ∼1 week (within-subject design). PANAS and word recognition memory ERP data are reported in Chow et al. (2021). Here, we report on auditory data (pure-tone audiogram, QuickSIN, speech-ERPs/FFRs) and their relation to changes in working memory scores pre-to-post stimulation.

We used a counterbalanced, crossover (repeated-measures) design in which participants underwent three testing sessions each featuring a different neurostimulation condition. The entire protocol, including measures reported previously in Chow et al. (2021) is summarized in Figure 1. Sessions were conducted ∼1 week apart scheduled at the same time of day to prevent carry-over effects (Thair et al., 2017) and circadian influences on performance (Krause and Cohen Kadosh, 2014; Li et al., 2015). In the *tDCS-only* condition, participants received anodal tDCS in silence. In the *tDCS*+*Music* condition, participants received anodal tDCS while simultaneously listening to autobiographically salient music. In the *Sham*+*Music* condition, participants received sham tDCS stimulation while simultaneously listening to autobiographically salient music. Critically, participants were blind to session type (although participants were no doubt aware of the presence/absence of music). Sessions were counterbalanced across participants.

During the first session, participants completed demographic questionnaires, audiometric testing, and the QuickSIN test (Killion et al., 2004) to ensure normal hearing thresholds and assess complex (i.e., speech-in-noise) listening abilities. Participants then completed measures of self-reported emotional affect (PANAS; Watson et al., 1988) and WM. The latter was assessed pre- and post-tDCS via the forward and backward digit spans tests from the Wechsler Memory Scale (WMS-III) (Wechsler, 1997). Both the PANAS and digit span tests were assessed pre- and post-tDCS.

The stimulation phase followed, involving one of *tDCS-only*, *tDCS*+*Music*, *or Sham*+*Music* conditions. In the conditions involving music listening, each song from the personalized playlist was played in its entirety. The order of songs was randomized for each participant’s playlist between the tDCS+Music and Sham+Music sessions. Autobiographically salient music was played through soundbooth speakers at an individual comfortable listening level that was kept constant for each participant’s two music listening conditions. Immediately following tDCS, participants completed another measure of emotional affect and WM.

After behavioral assays and a word recall EEG task (25 min) [reported in Chow et al. (2021)], participants completed EEG testing in which we recorded auditory ERPs and FFRs to a train of rapid speech phonemes. Individual tokens were a 100-ms English consonant-vowel /ba/ from the UCLA Nonsense Syllable Test (Dubno and Schaefer, 1992). The mean fundamental frequency (F0) was 150 Hz. Other acoustic characteristics are reported elsewhere (Bidelman et al., 2019). A total of 2,400 trials (alternating polarity; SOA = 250 ms) were collected. This same speech token was used to record both ERPs and FFRs simultaneously (e.g., Bidelman et al., 2013). Stimuli were presented binaurally through ER-3A insert earphones (Etymotic Research Inc., Elk Grove, IL, United States) at 76 dB SPL (A-weighted). Participants completed the remaining stimulation conditions on separate visits with the same procedure but without the initial hearing assessments from the first study visit. All testing sessions were conducted while the participant was seated comfortably in a recliner inside an electromagnetically shielded double-walled sound-attenuated booth.

### Neurostimulation

**FIGURE 2:**
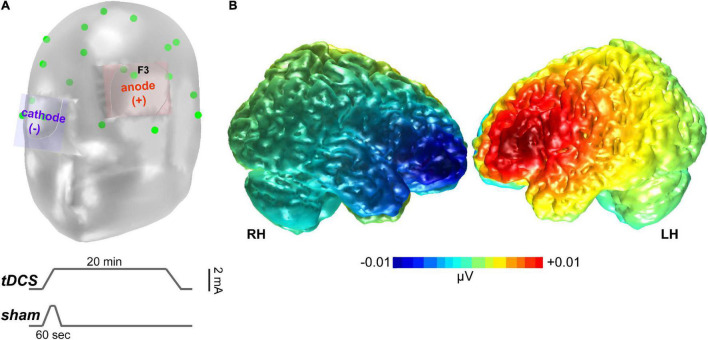
tDCS stimulation. **(A)** Montage of the stimulating electrodes. Green points show standard 10–20 electrode locations Lower traces show the time course (not to scale) of current stimulation for actual (tDCS) vs. sham sessions. **(B)** COMETS2 simulation (Lee et al., 2017) of the tDCS-induced current activation that targeted dorsolateral prefrontal cortex.

Transcranial direct current stimulation was performed in accordance with the relevant guidelines and regulations, including safety protocols based on established guidelines on tDCS administration (Rossi et al., 2009). A constant direct current (2 mA, 20 min) was administered by a battery-driven constant current stimulator from TCT Research (TCT Research Limited, Kowloon, Hong Kong) through 35 cm^2^ saline-soaked synthetic sponge electrodes (Figure 2A). The anode was placed over the left DLPFC, located over the F3 electrode location according to the International 10–20 system of EEG electrode placement. Evidence for using a F3 anode for targeting the left DLPFC is supported by prior studies (Seibt et al., 2015). The cathode was placed over the contralateral supraorbital region. We further confirmed the foci of stimulation via model simulations in the COMETS2 toolbox (Lee et al., 2017), which showed substantial current flow across DLPFC under this montage (Figure 2B).

#### Transcranial Direct Current Stimulation

Actual neurostimulation lasted 21 min. At the start of the stimulation phase, the current gradually increased in a ramp-like fashion from 0 to 2 mA over 60 s, remained constant for 19 min and 50 s, then was ramped down to 0 mA over 10 s (Figure 2A).

#### Sham

Current also gradually increased to 2 mA over a time window of 60 s, then immediately ramped down to 0 mA over 10 s period where it was maintained at that level of current for 20 min until the end of the stimulation phase. To ensure that no participant experienced adverse side effects, a post-stimulation questionnaire was administered that solicited listeners’ perception of several side effects (Likert scale: “0” being absent, “4” being severe).

### Electroencephalography Recording and Preprocessing

Neuroelectric brain activity (EEG) was recorded from eight electrodes (Fpz, Fz, Cz, Pz, P3/4, and TP9/10) at standard 10–20 locations on the scalp (Oostenveld and Praamstra, 2001) using BioSemi ActiveTwo amplifiers (BioSemi V.O.F., Amsterdam, Netherlands). This system used a pair of common mode sense and driven right leg reference electrodes. EEGs were digitized online at 16,384 Hz. EEG pre-processing was done using the Brainstorm MATLAB toolbox (Tadel et al., 2011). Traces were then downsampled to 5,000 Hz and re-referenced to linked mastoids (i.e., average of TP9/10 electrodes) for subsequent ERP analysis. Ocular artifacts (saccades and blinks) were corrected in the continuous EEG using a principal component analysis (Picton et al., 2000). Cleaned EEGs were then bandpass filtered (1–40 Hz), epoched (−50–200 ms), baseline corrected to the pre-stimulus period, and trial-wise averaged to obtained speech-evoked ERPs for each tDCS session per listener. ERPs and scalp topographies were visualized using the MNE toolbox (v0.23) (Gramfort et al., 2013) and BESA^®^ Research 7.1 (BESA, GmbH).

### Cluster-Based Permutation Analysis (Topographic ANOVA)

We used cluster-based permutation statistics (Maris and Oostenveld, 2007) to test for session-related changes in the cortical ERPs. A two-stage analysis was conducted in BESA Statistics 2.1 (BESA, GmbH). First, we computed an omnibus repeated measures (rm) ANOVA (*F*-test) contrasting ERP amplitudes between sessions at every time sample in the epoch window. This preliminary step identified contiguous clusters of data points both in time (adjacent samples) and electrodes where responses differed between tDCS sessions (i.e., fell below the cluster-building alpha, *p* < 0.05). For each spatiotemporal cluster, the sum of *t*-values of sampling points within each cluster then form the cluster-level statistics (or so called “cluster values”). In the subsequent analysis phase, permutation testing was then conducted using a Monte-Carlo resampling technique, which involved comparing the observed cluster value with random cluster values drawn from a permutation distribution. This permutation distribution was created by randomly assigning levels of the factor of interest (here, session) and iteratively conducting the same test (*N* = 1,000 resamples), retrieving the maximum cluster value for each permutation. The largest absolute cluster value is subsequently compared to the permutation distribution of maximal cluster values. If the maximum cluster value from the observed data is larger than 95% of the maximum cluster values in the permutation distribution, then the null hypothesis (i.e., that the sessions are sampled from the same distribution) is rejected. This identified significant *post hoc* differences by permuting between session conditions (e.g., Oostenveld et al., 2011). These contrasts were corrected with Scheffe’s test, using Holm–Bonferroni adjustments. This identified contiguous time samples for which the session conditions were not interchangeable. Importantly, this clustering process corrects for multiple comparisons across the aggregate of all time points and electrodes by controlling the familywise error rate. For an in-depth overview of permutation statistics as implemented in BESA Statistics, see Maris and Oostenveld (2007).

### Brainstem Frequency-Following Responses

Our high sample rate allowed us to recover the FFR, a neurophonic response that reflects phase-locked activity to speech with dominant sources in the brainstem when recorded via EEG (Bidelman, 2018; Ross et al., 2020). To this end we measured the root mean squared (RMS) amplitude of brainstem FFRs, computed during the steady-state portion (20–120 ms) of the response waveform. FFRs were analyzed at the Fz electrode, referenced to average mastoids (i.e., Fz – TP9/TP10). This montage is optimal for recording FFRs of midbrain origin (Bidelman, 2015). FFR RMS amplitudes were analyzed via a one-way mixed-model ANOVA (fixed factor = session type; subjects = random effect) in R (lme4 package; Bates et al., 2015).

## Results

### Brainstem Frequency-Following Responses

**FIGURE 3:**
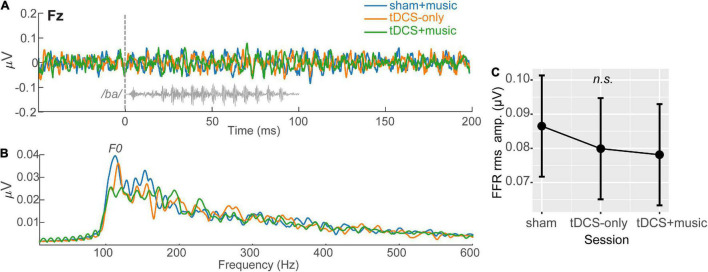
Speech-evoked FFRs (brainstem responses) across tDCS sessions. Grand averaged FFRs (mean of 2400 trials per participant) are shown at electrode Fz, referenced to linked mastoids (i.e., Fz – TP9/10 montage). **(A)** FFR time waveforms and **(B)** response spectra. FFR F0, corresponding to the voice pitch of the speech stimulus (/ba/) is demarcated in the FFRs.**(C)** FFR RMS showed a trend for decreasing amplitudes with tDCS but the session effect was not significant (*p* = 0.61). Error bars = 95% CI.

Frequency-following responses are shown in Figure 3. Recent studies have shown FFRs are modulated when tDCS stimulation is applied over the right temporal lobe (Mai and Howell, 2021). A one-way ANOVA including the factor tDCS session (tDCS+Music; tDCS alone; Sham+Music) and the dependent variable FFR RMS amplitude indicated FFRs were invariant across tDCS sessions [*F*(2,26) = 0.49, *p* = 0.61] (Figure 3C). Given the poor morphology and null session effects, we did not pursue FFRs further and focus only on analysis of the cortical ERPs hereafter.

### Cortical Auditory Event-Related Potentials

**FIGURE 4:**
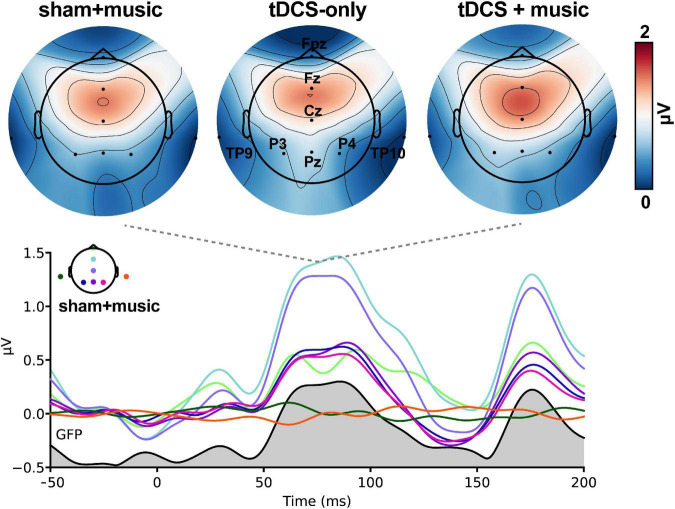
Speech-evoked ERPs (cortical responses) across tDCS sessions. Hot colors, increased positive voltage. **(Top)** Maps are shown at a latency of 75 ms, the approximate latency of the P1 wave. **(Bottom)** Butterfly overlay of the auditory ERPs (only the *Sham*+*music* condition is shown for clarity). Channel colors in the bottom panel correspond with the electrode names marked in the top middle heatmap. Gray area = global field power (GFP).

Speech-evoked cortical responses are shown as topographic maps and global field power in Figure 4. Prominent activity was observed within 200 ms of stimulus onset corresponding to the canonical P1-N1-P2 deflections of the auditory ERPs.

**FIGURE 5:**
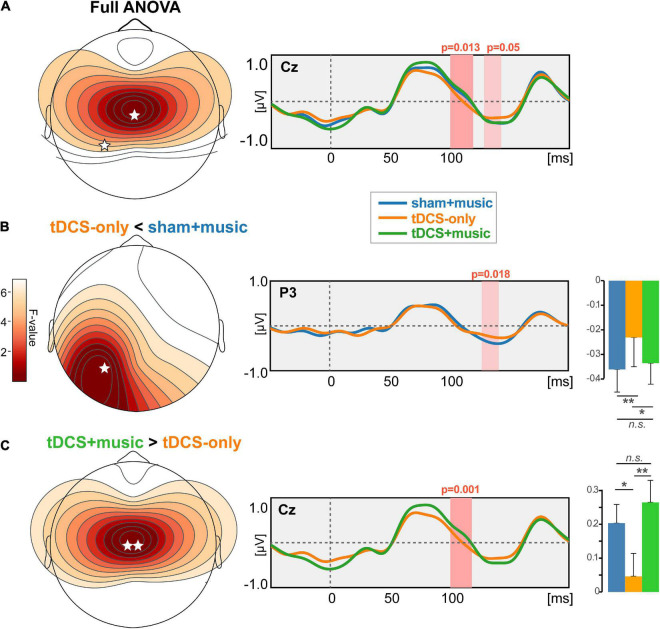
tDCS and tDCS+music differentially alters auditory cortical responses to speech. **(A)** Results of a cluster-based permutation testing (omnibus ANOVA) comparing ERPs across sessions. Speech ERPs are modulated in the 100–150 ms time window depending on session type. **(B)** *Post hoc* effect contrasting tDCS-only vs. Sham+music. Music listening (in the absence of tDCS) yielded stronger speech responses in 150 ms timeframe compared to tDCS stimulation alone. **(C)** *Post hoc* effect contrasting tDCS+music vs. tDCS alone. Whereas tDCS suppressed the auditory ERPs, concurrent music listening seemingly counteracted the suppressive effect of tDCS on neural responses. **p < 0.01, *p < 0.05, error bars = ±1 SEM.

The topographic omnibus ANOVA revealed session-related modulations in the timeframe of the N1 wave (100–150 ms; Figure 5A). Differences were most prominent at the vertex where the auditory ERPs are maximal on the scalp surface. However, cluster-based permutation testing (corrected for multiple comparisons across time samples and electrodes) revealed this session effect was attributable to modulations within two time windows. The first cluster from 126 to 141 ms showed weaker (i.e., less negative) N1 responses over the P3 electrode in the tDCS-only vs. Sham+music condition (*p* = 0.018, Figure 5B). The second cluster spanning 100–118 ms showed larger (i.e., more positive) responses at Cz for the tDCS+music compared to the tDCS-only condition (*p* = 0.001; Figure 5C). Collectively, these findings reveal tDCS and/or music (or a combination of both) modulate in the early cortical processing of speech. Whereas tDCS alone generally weakened neural responses, concurrent music restored responses to nominal activation levels similar to the Sham+Music condition (without tDCS).

### Brain-Behavior Relations

**FIGURE 6:**
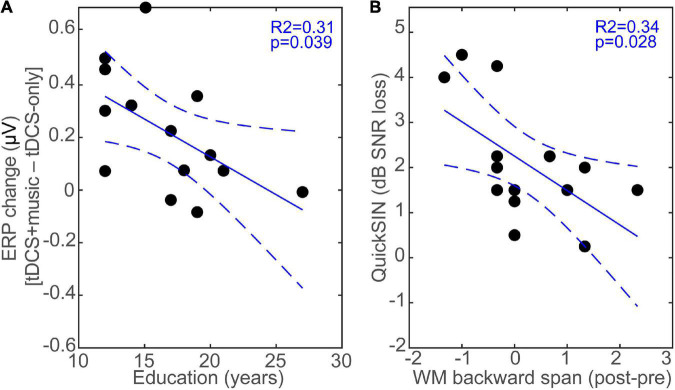
tDCS induced changes in neuro-behavioral responses depend on education and relate to speech in noise listening skills. **(A)** The music-induced changes during tDCS above and beyond neurostimulation alone (i.e., *tDCS*+*music* – *tDCS-only)* is negatively related to education; listeners with more educational attainment experience less music-induced change in the ERPs during tDCS. **(B)** Improvements in WM (backward digit span) pre-to-post tDCS stimulation is related to better SIN listening skills. Pre/post test scores are collapsed across sessions. Dotted lines = 95% CI.

We assessed the behavioral relevance of the ERP changes due to tDCS as well as relations between behavioral and demographic variables via bivariate linear regressions (“fitlm” in MATLAB). Shapiro–Wilk tests confirmed normality of the variables (all *P*s > 0.064). We focused on auditory measures including hearing thresholds, speech-in-noise processing (QuickSIN), and working memory (digit span) performance, as these factors are important determinants of everyday speech recognition abilities in older adults (e.g., Killion et al., 2004; Moore et al., 2014; Füllgrabe and Rosen, 2016a,b; Bidelman et al., 2019; Vermeire et al., 2019; Yeend et al., 2019). We computed the music-induced modulation of the ERP during tDCS above and beyond tDCS neurostimulation alone as the difference in ERP response amplitudes identified via the permutation statistics (i.e., *tDCS*+*music* – *tDCS-only;* see bars in Figure 5C) and regressed this ERP measure against each of the behavioral and demographic measures. Analysis showed that the tDCS+music changes in ERP amplitude was not associated with QuickSIN, PTA thresholds, nor WM performance (all *p*s > 0.05; data not shown). Similarly, a linear model incorporating the combination of variables shown in Figure 6 did not reveal any interaction or multivariate factors as significant predictors.

Because educational level moderates some of the benefits from tDCS (Berryhill and Jones, 2012; Krebs et al., 2020), we further evaluated correlations between tDCS neuromodulation and listeners’ education. We found a negative association between education attainment and the music-related modulation in ERPs during tDCS; listeners with more formal education showed lower neuroplastic change in the N1 than those with lesser education (*R*^2^ = 0.31, *p* = 0.039; Figure 6A). Additionally, performance on the QuickSIN averaged 2.09 ± 1.31 dB SNR loss, consistent with scores in this age bracket (Zendel and Alain, 2012; Hutka et al., 2013; Bidelman et al., 2019). While QuickSIN was not correlated with neural measures *per se* (all ERPs were evoked by clean speech), we did find that the change in WM^1^ performance pre- to post-tDCS (averaged across sessions) was associated with QuickSIN scores. Namely, larger behavioral gains in backward digit span pre-to-post tDCS were associated with better speech-in-noise recognition (i.e., lower QuickSIN scores) (*R*^2^ = 0.34, *p* = 0.028; Figure 6B).

## Discussion

We assessed whether combining anodal tDCS over DLPFC paired with listening to autobiographically salient music alters neural speech processing in older adults compared to either music listening under sham stimulation or tDCS alone. Our findings reveal tDCS and/or music induced modulations in the early neural encoding of speech between 100 and 150 ms after stimulus onset. Whereas tDCS effects appeared largely suppressive (i.e., reducing ERP amplitude as compared to Music+sham), concurrent music with tDCS restored ERP amplitude to baseline levels. However, we note this neural effect could be attributable to a true effect of tDCS or presence/absence music. Changes in WM were also observed from pre- to post intervention session, and were related to better speech-in-noise listening skills. Our findings provide new causal evidence that tDCS stimulation modulates the early neural encoding of speech as indexed by the auditory ERPs. Moreover, we show that tDCS-related improvements in WM [previously reported in Chow et al. (2021)] are also related to noise-degraded speech perception in older adults.

In the same sample of listeners, we have previously shown that tDCS while listening to autobiographically salient music amplifies gains in WM relative to either tDCS or music listening alone (Chow et al., 2021). Several studies have reported tDCS-related improvements in WM for younger adults (Andrews et al., 2011; Jeon and Han, 2012) but similar effects in older adults have been equivocal. Moreover, to our knowledge, only one other study (Mansouri et al., 2017) has examined effects of anodal tDCS on executive functioning by measuring response inhibition in younger adults pre- and post-tDCS in the presence of background music. That study found that high-tempo background music interacts with tDCS on response inhibition. Our dataset extends this work to the domain of WM and healthy older adults. We also extend our previous findings on the effects of tDCS on older adults’ memory recognition (Chow et al., 2021) by demonstrating tDCS over DLPFC has a modulatory effect on the neural encoding of speech sounds.

Literature on the effects of tDCS on the auditory ERPs have been equivocal (for review, see Wang et al., 2020). Heimrath et al. (2016) found anodal tDCS over temporal lobe increased the P1 (∼50 ms) response to consonant-vowel speech tokens similar to those used here. However, other studies have failed to observe significant ERP changes with similar tDCS stimulation protocols (Kunzelmann et al., 2018; López-Caballero et al., 2020). Our study differs from prior work in that our stimulation targets were located over frontal (rather than auditory temporal) cortical sites. We found that tDCS suppressed the auditory ERPs in time range of the N1, but that concurrent music reinvigorated neural responses and seemingly counteracted the suppressive tDCS effect. By stimulating DLPFC, we therefore find small but measurable tDCS-induced changes in the brain’s early auditory-cortical encoding of speech. Presumably, these DLPFC changes in auditory responses result from the top-down functional projections from prefrontal areas to auditory cortex (Hackett et al., 1999; Plakke and Romanski, 2014). Moreover, we note that EEG was recorded ∼25 min after the neurostimulation sessions. Thus, the observed effects might actually *underestimate* the true neuroplastic effects had FFRs/ERPs been conducted immediately after tDCS intervention. Still, even after 25 min, tDCS effects on the brain appear to persist long enough to observe modulations in the cortical ERPs (but not brainstem FFRs) post-stimulation.

What might be the mechanistic account of these data? The putative generator(s) of the N1 are thought to lie near the auditory cortices within the Sylvian fissure (Näätänen and Picton, 1987; Scherg et al., 1989; Picton et al., 1999). Functionally, N1 is associated with the formation of perceptual object representations and auditory feature coding (Näätänen and Picton, 1987; Alain and Arnott, 2000). The auditory N1 also receives top-down influences from prefrontal brain regions (Chao and Knight, 1997; Knight et al., 1999) and is often exaggerated with aging, which has been taken as evidence for reduced inhibition from distal (frontal) areas (Chao and Knight, 1997; Alain et al., 1999, 2022; Caspary et al., 2008; Bidelman et al., 2014). More robust neural connectivity between frontal and temporal cortices is also related to better speech-in-noise processing in older adults (Price et al., 2019). Presumably, the largely suppressive tDCS-related changes in N1 observed here may result from similar efferent mechanisms, by which increased involvement of the frontal cortices due to anodal (excitatory) stimulation of DLPFC (Thair et al., 2017) modulates the early sensory encoding of speech sounds. Alternatively, we cannot rule out the possibility that the effects of tDCS on the auditory ERPs might result from other mechanisms. For instance, the N1 is modulated by attention and arousal state (Hillyard et al., 1973; Coull, 1998); thus, it is conceivable that the observed tDCS changes in auditory responses may have resulted from subtle (covert) changes in attention to the speech stimuli. However, we note our ERP paradigm featured strictly passive listening, so this explanation appears insufficient.

Transcranial direct current stimulation did not have an appreciable effect on speech-evoked FFRs. We offer several explanations for these null findings. First, FFRs are dominantly of brainstem origin when recorded via EEG (Bidelman, 2018; Ross et al., 2020). As such, the response may be too deep (peripheral) in the brain to show sensitivity to distal scalp stimulation. Relatedly, our tDCS paradigm targeted frontal lobe sites. Thus, the orientation and location of intracranial current densities induced by tDCS were not optimized for neuromodulation of lower auditory brain areas (i.e., brainstem). However, we note that even with more optimal foci for auditory neuromodulation (e.g., superior temporal lobe), the effects of magnetic (TMS) and current (tDCS) stimulation on FFRs remains equivocal (López-Caballero et al., 2020; Mai and Howell, 2021). For example, continuous theta burst TMS over right auditory cortex fails to yield measurable changes in FFRs suggesting deeper, more peripheral sources (i.e., brainstem) which are insensitive to cortical stimulation (López-Caballero et al., 2020). In contrast, Mai and Howell (2021) showed that tDCS neurostimulation over right auditory cortex can cause subtle decreases in speech-FFR amplitudes (i.e., F0 coding), though apparently without concomitant changes in pitch discrimination behavior (Mai and Howell, 2021). Thus, it is possible that more sessions or more targeted, high-definition (i.e., focal) tDCS stimulation sites proximal to auditory cortex may have been more successful in altering FFRs, either directly by altering phase-locked FFR activity stemming from auditory cortex and/or top-down modulation of brainstem FFR components via corticofugal efferent pathways (e.g., Price and Bidelman, 2021). Lastly, our stimulus design may have weakened possible tDCS-FFR effects. Indeed, there is some suggestion that binaural stimulus presentation (as used here) might smear tDCS-induced changes in FFR, which are more prominent for monaural (contralateral) stimulus delivery (Mai and Howell, 2021). Our consonant-vowel speech tokens evoke weaker FFRs given their short duration of their vowel periodicity (<40 ms) (Bidelman et al., 2019). Future studies with more optimal FFR-evoking stimuli (e.g., sustained vowels) and stimulation sites (e.g., temporal lobe) are needed to fully evaluate the effects of tDCS on speech-FFRs (cf. Mai and Howell, 2021).

Our correlational findings offer preliminary evidence for tDCS-induced modulation of cognitive skills in the form of better WM post-intervention [as also reported in Chow et al. (2021)]. Notable in present study, we show these WM improvements attributable to tDCS may have a potential transfer effect, bolstering degraded speech perception performance. Still, since we only collected baseline QuickSIN scores, a way to directly test this hypothesis in future studies would be to compare pre- and post-tDCS stimulation QuickSIN performance. Difficulty understanding speech-in-noise is highly prevalent among the aging population and older adults exhibit greater listening effort in adverse listening situations (e.g., Helfer and Wilber, 1990; Wong et al., 2010). We found pre- to post-tDCS session changes in WM were correlated with better (i.e., lower threshold) QuickSIN performance. WM capacity is highly predictive of degraded speech-listening skills in younger adults, and especially in older adults (e.g., Füllgrabe and Rosen, 2016a,b; Alain et al., 2018; Vermeire et al., 2019; Yoo and Bidelman, 2019; Bidelman and Yoo, 2020). Thus, our behavioral data suggest tDCS paired with music listening may be a viable intervention to boost cognitive and WM performance, and in turn, receptive communication skills that decline during the lifespan. Still, the generalizability of these findings may be limited by the high level of education and greater number of females in our cohort. Indeed, there is some indication that responsivity to tDCS is larger in females than males (Dedoncker et al., 2016). Although our sample size was similar to those of past tDCS and music listening studies (Picazio et al., 2015; Mansouri et al., 2017), we note our dataset (Chow et al., 2021) is small in size, which is a limitation of our study. Still, our data show that the degree of neuroplasticity induced by tDCS+Music is correlated with listeners’ years of formal education. Consequently, those with less scholastic achievement may have “more to gain” from tDCS interventions. tDCS effects are also variable across cognitive domains and are highly dependent on stimulation parameters (e.g., stimulation current intensity, duration, and site) (Huo et al., 2021). Additionally, future studies could assess whether the type (genre, content) and degree of familiarity of music listeners self-select differentially alters auditory responses (cf. Brown and Bidelman, 2022). Our design also did not include a tDCS sham *without* music listening, which would have allowed for more robust inference regarding the independent effects of tDCS and music on the auditory ERPs. There is a limitation of our study which warrants future investigation. Longitudinal and larger sample studies are also needed to identify the proper dosage of neurostimulation that might produce optimal neuro-behavioral plasticity at the individual level.

We should also emphasize that the present data did not show a link between neural and behavioral outcomes. That is, we did not observe correlations between tDCS+music benefits and auditory measures including QuickSIN, hearing thresholds, and WM performance. Thus, like our previous report examining tDCS and recognition memory (Chow et al., 2021), the behavioral relevance of our findings remains open. It is possible the effects of tDCS+music pairing are indirect and somewhat epiphenomenal. Indeed, TMS stimulation of left DLPFC increases musical reward sensitivity through fronto-striatal pathways (Mas-Herrero et al., 2018). tDCS modulation of these pathways may heighten arousal from music listening and thereby also heighten the effects of cognitive performance and auditory processing as observed here. Pairing tDCS with personally meaningful music may also make the experience of tDCS more tolerable and enjoyable, allowing more focused task performance after stimulation (see Chow et al., 2021).

Regardless of underlying mechanism(s), our study affirms that listening to music, particularly to songs that evoke autobiographical memories, may heighten responsivity to the effects of tDCS for healthy older adults. The larger effect of music+tDCS than music or tDCS alone supports general findings in the literature of an advantage of combining tDCS with other cognitive tasks to maximize neuroplastic effects (e.g., Assecondi and Shapiro, 2018). Moreover, we demonstrate tDCS over DLPFC causes small but measurable changes in neuroplasticity related to WM and the neural encoding of speech sounds. These neurobehavioral changes may have indirect benefits to speech-in-noise processing in older adults. Our findings help inform future work aimed at tailoring personalized neurostimulation interventions in older adults to bolster cognitive skills and complex auditory processing.

## Data Availability Statement

The raw data supporting the conclusions of this article will be made available by the authors, without undue reservation.

## Ethics Statement

The studies involving human participants were reviewed and approved by the Research Ethics Board of the Rotman Research Institute at Baycrest Centre. The participants provided their written informed consent to participate in this study.

## Author Contributions

AN-G, JR, and CA contributed to the conception and design. AN-G and RC conducted the data acquisition. RC, KB, and GB conducted the data processing and analysis. All authors contributed to interpreting the results and writing the manuscript.

## Conflict of Interest

The authors declare that the research was conducted in the absence of any commercial or financial relationships that could be construed as a potential conflict of interest.

## Publisher’s Note

All claims expressed in this article are solely those of the authors and do not necessarily represent those of their affiliated organizations, or those of the publisher, the editors and the reviewers. Any product that may be evaluated in this article, or claim that may be made by its manufacturer, is not guaranteed or endorsed by the publisher.

## Footnotes

1 As reported in Chow et al. (2021), an ANOVA conducted on digit span backward WM scores showed a tDCS session × time interaction [*F*(2,26) = 4.12, *p* = 0.028]. Pairwise comparisons revealed a significant increase in WM scores pre-to-posttest in the tDCS+Music condition (*p* = 0.012) but no change in performance for the tDCS-only (*p* = 0.671) or Sham+Music (*p* = 0.418) conditions.
